# Levosimendan use in patients with acute heart failure and reduced ejection fraction with or without severe renal dysfunction in critical cardiac care units: a multi-institution database study

**DOI:** 10.1186/s13613-021-00810-y

**Published:** 2021-02-08

**Authors:** Cze-Ci Chan, Kuang-Tso Lee, Wan-Jing Ho, Yi-Hsin Chan, Pao-Hsien Chu

**Affiliations:** Department of Cardiology, Chang Gung Memorial Hospital, College of Medicine, Chang Gung University, Taoyuan, Taiwan

**Keywords:** Levosimendan, Dobutamine, Heart failure, Renal failure

## Abstract

**Background:**

Acute heart failure is a life-threatening clinical condition. Levosimendan is an effective inotropic agent used to maintain cardiac output, but its usage is limited by the lack of evidence in patients with severely abnormal renal function. Therefore, we analyzed data of patients with acute heart failure with and without abnormal renal function to examine the effects of levosimendan.

**Methods:**

We performed this retrospective cohort study using data from the Chang Gung Research Database (CGRD) of Chang Gung Memorial Hospital (CGMH). Patients admitted for heart failure with LVEF ≤ 40% between January 2013 and December 2018 who received levosimendan or dobutamine in the critical cardiac care units (CCU) were identified. Patients with extracorporeal membrane oxygenation (ECMO) were excluded. Outcomes of interest were mortality at 30, 90, and 180 days after the cohort entry date.

**Results:**

There were no significant differences in mortality rate at 30, 90, and 180 days after the cohort entry date between the levosimendan and dobutamine groups, or between subgroups of patients with an estimated glomerular filtration rate (eGFR) ≥ 30 mL/min/1.73 m^2^ and eGFR < 30 mL/min/1.73 m^2^ or on dialysis. The results were consistent before and after propensity score matching.

**Conclusions:**

Levosimendan did not increase short- or long-term mortality rates in critical patients with acute heart failure and reduced ejection fraction compared to dobutamine, regardless of their renal function. An eGFR less than 30 mL/min/1.73 m^2^ was not necessarily considered a contraindication for levosimendan in these patients.

## Background

Acute heart failure (AHF) is a life-threatening condition defined as a status of acute-onset or rapidly worsening heart failure symptoms. The clinical spectrum of heart failure is wide, ranging from mild pulmonary congestion to cardiogenic shock. Patients with AHF and low cardiac output are at a higher risk of morbidity and mortality [1]. For critical patients with AHF and systemic hypoperfusion, inotropic agents or mechanical cardiac support devices are used to maintain hemodynamic stability. Medications including vasopressors and sympathomimetic inotropes such as norepinephrine, dopamine, dobutamine, and levosimendan are frequently used to treat AHF with reduced LVEF to improve cardiac output. Levosimendan is a Ca^2+^-sensitizer of the myocardium, which induces vasodilatation and increases cardiac output [2–4]. It is particularly effective in patients with acute decompensated heart failure by improving left ventricular ejection fraction (LVEF), decreasing B-type natriuretic peptide levels, improving renal function, and decreasing the need for renal replacement therapy in different settings [5]. Previous studies have demonstrated a comparable effect on improving cardiac output in heart failure patients to dobutamine, and it may result in a greater improvement in glomerular filtration rate than dobutamine in patients with cardiorenal syndrome [6–12]. However, evidence supporting the use of levosimendan in patients with severe renal dysfunction (i.e., an estimated glomerular filtration rate (eGFR) of < 30 mL/min/1.73 m^2^) is limited, particularly in patients with AHF and low cardiac output. In this study, we hypothesized that severe renal dysfunction may frequently be complicated with AHF, and that levosimendan may be beneficial in these patients. Therefore, the aim of this study was to analyze patients with AHF and reduced LVEF with or without abnormal renal function to examine the effects of levosimendan.

## Methods

### Data source

We performed this retrospective cohort study using data from the Chang Gung Research Database (CGRD) of Chang Gung Memorial Hospital (CGMH), which includes two medical centers, two regional hospitals, and three district hospitals around Taiwan. The total numbers of outpatient, emergency room and inpatient visits in 2018 in Taiwan were 420,283, 20,252 and 3172 per day, respectively [13]. Among the hospitals in Taiwan, CGMH has the largest volume of both inpatient (12.4%) and outpatient (21.2%) services in Taiwan. The CGRD comprises patient data derived from initial electronic medical records, and it was established for administrative and insurance purposes for CGMH. Disease diagnoses were coded using International Classification of Disease, Ninth Revision, Clinical Modification codes before 2016, and International Classification of Disease, Tenth Revision codes thereafter. Previous studies have provided additional detailed information on the CGRD [14, 15]. The Institutional Review Board of the Chang Gung Medical Foundation approved this study.

### Study design

Patients admitted for heart failure with decreased EF (defined as LVEF ≤ 40% by echocardiography) between January 2013 and December 2018 who received levosimendan or dobutamine in the critical cardiac care units (CCU) were identified from the CGRD. Patients who received levosimendan during the hospital stay were classified as the levosimendan group, regardless of whether they received dobutamine or other inotropic agents prior to the administration of levosimendan. All patients were prescribed inotropes because of heart failure with low cardiac output and systemic hypoperfusion (i.e., cardiogenic shock). Patients in the levosimendan group received continuous intravenous levosimendan at a dose of 0.05–0.2 µg per kilogram of body weight per minute for 72 h (without a loading dose). The date of cohort entry in the levosimendan group was defined as the day of the first levosimendan infusion during the hospital stay. To eliminate immortal time bias, the cohort entry date of the patients in the dobutamine group was assigned based on their counterparts in the levosimendan group [33]. Patients in the levosimendan group were matched with those in the dobutamine group by sex, age, eGFR, and LVEF. We excluded patients with extracorporeal membrane oxygenation (ECMO). The remaining patients were further classified according to their renal function using plasma creatinine and Modification of Diet in Renal Disease (MDRD) eGFR [16].

### Covariates and outcomes

We analyzed the following covariates: age, sex, LVEF, eGFR, inotropic agent use during the admission, acute myocardial infarction (AMI) or percutaneous coronary intervention (PCI) during admission, length of intensive care unit stay, hospital days, comorbidities (atrial fibrillation, diabetes mellitus, hypertension and dyslipidemia), medications (aspirin, clopidogrel, ticagrelor, beta-blockers, angiotensin-converting enzyme inhibitors (ACEis), angiotensin II receptor blockers (ARBs), mineralocorticoid receptor antagonists (MRAs), digoxin, amiodarone and ivabradine), and baseline laboratory data (hemoglobin, alanine aminotransferase, B-type natriuretic peptide, HCO3, total bilirubin, blood urea nitrogen, sodium, potassium, platelets, hematocrit, white blood count, albumin, lactate and international normalized ratio). All patients underwent transthoracic echocardiography in the emergency department or after they had been admitted to a CCU. We followed the guidelines of the American Society of Echocardiography and used the biplane method to calculate the LVEF. The patients were defined as having a comorbidity if they had at least two outpatient diagnoses or one inpatient diagnosis for that comorbidity prior to the cohort entry date. Medication and laboratory data for 3 months prior to the cohort entry date were extracted. The outcomes of interest were mortality at 30, 90, and 180 days after the cohort entry date. We also evaluated in-hospital mortality after dosing. Patient follow-up was censored 6 months after the cohort entry date, last visit at CGMH, date of death, or December 31, 2018, whichever occurred first.

### Statistical analysis

We evaluated differences in baseline characteristics between groups using standardized differences (STDs), where an absolute value of > 0.2 was considered to be a substantial difference. We performed propensity score matching (PSM) based on the propensity score derived from multivariable logistic regression to balance the two groups. Each patient in the levosimendan group was matched to two counterparts in the dobutamine group, if possible. Propensity scores were calculated using age, sex, LVEF, eGFR, use of inotropic agents (dopamine, norepinephrine, and epinephrine), AMI, myocarditis and mechanical ventilation during the index admission, intra-aortic balloon pumping, Sequential Organ Failure Assessment (SOFA) score and Acute Physiology and Chronic Health Evaluation III (APACHE III) score. PSM was performed separately for those with an eGFR ≥ 30 mL/min/1.73 m^2^ and those with an eGFR < 30 mL/min/1.73 m^2^ or dialysis. A univariate Cox proportional hazard model was used to compare the mortality rates between groups. Statistical significance was set at a two-sided *P* value of < 0.05, and no adjustment of multiple testing (multiplicity) was made in this study. We used SAS (version 9.4; SAS Institute, Cary, NC, USA) to perform all statistical analyses.

## Results

**Fig. 1:**
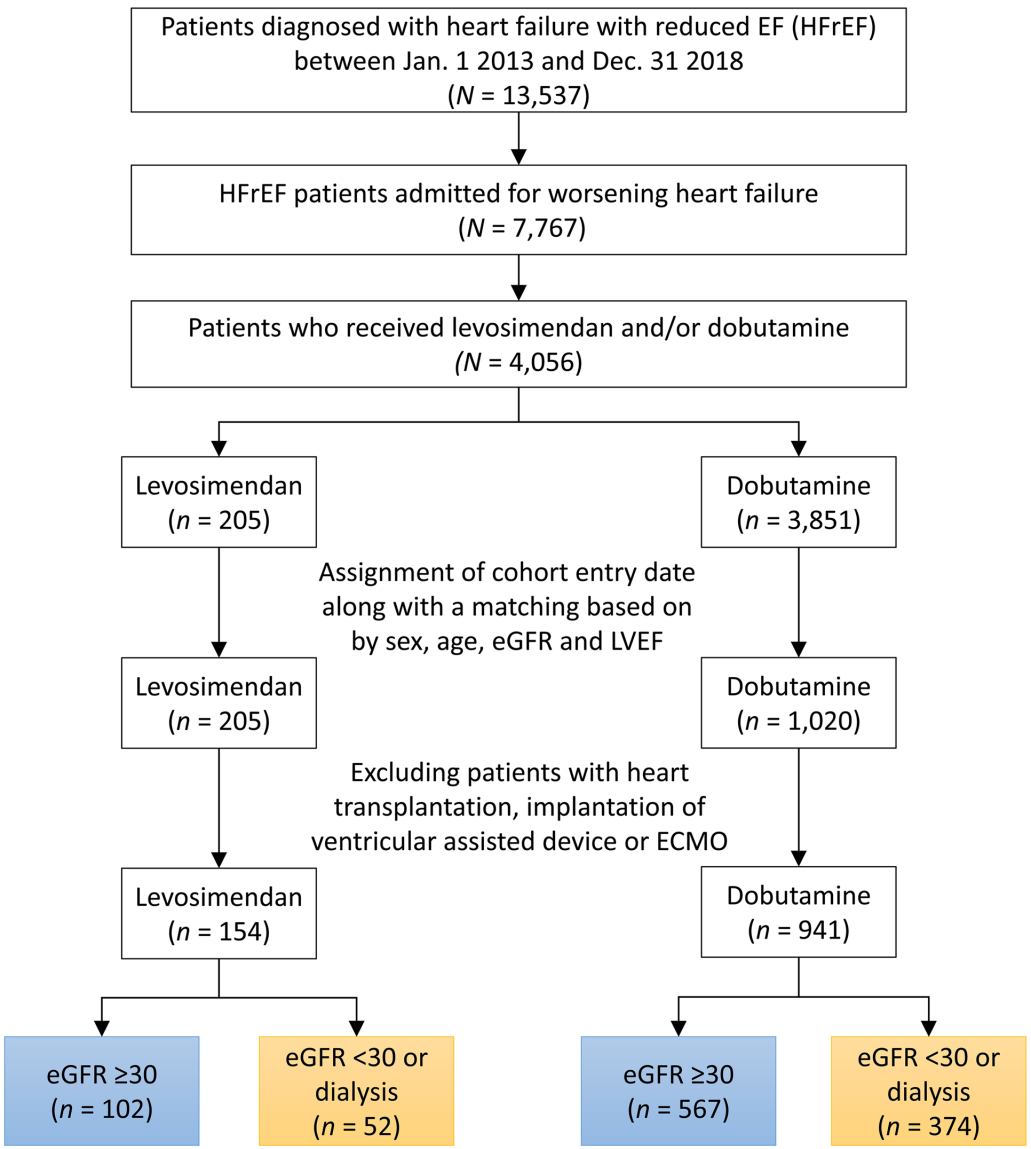
Study flowchart

After matching, 102 and 567 patients with eGFR > 30 mL/min/1.73 m^2^ were classified into the levosimendan and dobutamine groups, respectively, and 52 and 374 patients with eGFR ≤ 30 mL/min/1.73 m^2^ or dialysis were classified into the levosimendan and dobutamine groups (Fig. 1), respectively.

### Baseline characteristics (supplemental digital content)

Before PSM, patients in the levosimendan group were more likely to receive dopamine and less likely to receive epinephrine during hospital stay. The usage rate of norepinephrine was comparable. Myocarditis, AMI, PCI, and IABP were more common in the levosimendan group. After PSM, 63 patients in the levosimendan group with an eGFR ≥ 30 mL/min/1.73 m^2^ had 2 counterparts and 19 patients had only 1 counterpart, resulting in a total of 145 patients in the dobutamine group. After PSM, 38 patients in the levosimendan group with an eGFR < 30 mL/min/1.73 m^2^ or dialysis had 2 counterparts and 5 patients had only 1 counterpart, resulting in a total of 81 patients in the dobutamine group. No significant group differences were observed in age, sex, LVEF, eGFR, inotropic agent use, and AMI after PSM (Tables 1**, **2).

**Table 1:** Baseline characteristics of patients with eGFR ≥ 30 who received levosimendan versus dobutamine alone

Variable	Valid N	Before PSM^‡^			After PSM^‡^		
		Levosimendan (*n* = 102)	Dobutamine (*n* = 567)	STD	Levosimendan (*n* = 82)	Dobutamine (*n* = 145)	STD
Age, year*	669	65.8 ± 15.3	64.3 ± 15.1	0.10	65.5 ± 14.7	65.7 ± 15.6	− 0.01
Male*	669	76 (74.5)	437 (77.1)	− 0.06	64 (78.0)	105 (72.4)	0.13
LVEF, %*	669	28.7 ± 6.8	28.3 ± 7.8	0.05	27.7 ± 6.7	27.7 ± 7.8	< 0.01
eGFR, mL/min/1.73m^2^*	669	66.2 ± 31.9	65.8 ± 29.1	0.01	66.6 ± 32.5	67.6 ± 29.5	− 0.03
Inotropic agents during the index admission							
Dopamine*	669	24 (23.5)	67 (11.8)	0.31	15 (18.3)	24 (16.6)	0.05
Norepinephrine*	669	15 (14.7)	87 (15.3)	− 0.02	12 (14.6)	20 (13.8)	0.02
Epinephrine*	669	8 (7.8)	112 (19.8)	− 0.35	6 (7.3)	11 (7.6)	− 0.01
AMI during the index admission*	669	35 (34.3)	85 (15.0)	0.46	20 (24.4)	37 (25.5)	− 0.03
PCI during the index admission*	669	23 (22.5)	41 (7.2)	0.44	12 (14.6)	21 (14.5)	< 0.01
Myocarditis during the index admission*	669	6 (5.9)	8 (1.4)	0.24	3 (3.7)	2 (1.4)	0.15
Mechanical ventilator*	669	52 (51.0)	233 (41.1)	0.20	41 (50.0)	59 (40.7)	0.19
IABP*	669	34 (33.3)	26 (4.6)	0.79	14 (17.1)	20 (13.8)	0.09
ICU days	669	7 [4, 11]	2 [0, 6]	NA	7 [3, 10]	3 [0, 7]	NA
Admission days	669	19 [15, 36]	19 [12, 31]	NA	20 [15, 38]	18 [12, 29]	NA
Risk score*							
SOFA	669	6.4 ± 2.0	6.8 ± 2.2	− 0.20	6.4 ± 2.0	6.2 ± 1.8	0.10
APACHE III	669	37.5 ± 17.6	32.4 ± 16.2	0.31	37.3 ± 18.2	35.5 ± 19.0	0.10
Comorbidities							
Atrial fibrillation	669	39 (38.2)	216 (38.1)	0.00	35 (42.7)	47 (32.4)	0.21
Diabetes mellitus	669	44 (43.1)	266 (46.9)	− 0.08	38 (46.3)	85 (58.6)	− 0.25
Hypertension	669	63 (61.8)	391 (69.0)	− 0.15	51 (62.2)	107 (73.8)	− 0.25
Dyslipidemia	669	70 (68.6)	301 (53.1)	0.32	54 (65.9)	90 (62.1)	0.08
Baseline medications							
Aspirin	669	78 (76.5)	379 (66.8)	0.21	61 (74.4)	101 (69.7)	0.11
Clopidogrel	669	58 (56.9)	273 (48.1)	0.18	45 (54.9)	83 (57.2)	− 0.05
Ticagrelor	669	41 (40.2)	88 (15.5)	0.57	26 (31.7)	27 (18.6)	0.31
Beta-blockers	669	97 (95.1)	474 (83.6)	0.38	79 (96.3)	122 (84.1)	0.42
ACEi	669	65 (63.7)	314 (55.4)	0.17	49 (59.8)	79 (54.5)	0.11
ARBs	669	73 (71.6)	375 (66.1)	0.12	59 (72.0)	98 (67.6)	0.10
MRA	669	73 (71.6)	443 (78.1)	− 0.15	61 (74.4)	111 (76.6)	− 0.05
Digoxin	669	42 (41.2)	268 (47.3)	− 0.12	39 (47.6)	65 (44.8)	0.05
Amiodarone	669	72 (70.6)	298 (52.6)	0.38	60 (73.2)	60 (41.4)	0.68
Ivabradine	669	43 (42.2)	117 (20.6)	0.48	32 (39.0)	34 (23.4)	0.34
Baseline laboratory data							
Hemoglobin, g/dl	664	12.1 ± 2.5	11.8 ± 2.3	0.12	12.1 ± 2.5	11.8 ± 2.3	0.13
ALT, U/L	595	37 [23, 85]	25 [17, 50]	NA	35 [21, 96]	26 [18, 56]	NA
BNP, pg/mL	401	1451 [840, 3789]	1494 [772, 2560]	NA	1435 [840, 3810]	1751 [957, 2821]	NA
HCO3, mmol/L	289	23.6 ± 5.8	25.9 ± 5.7	− 0.40	23.9 ± 6.4	25.2 ± 5.1	− 0.22
Total bilirubin, mg/dL	413	1.7 ± 2.0	1.7 ± 2.1	0.01	1.7 ± 2.2	1.7 ± 2.7	0.00
BUN, mg/dL	647	30.3 ± 15.8	29.8 ± 16.6	0.03	30.0 ± 16.1	31.2 ± 19.9	− 0.07
Sodium, mg/dL	668	138.4 ± 5.3	139.6 ± 5.7	− 0.23	138.2 ± 5.1	139.4 ± 6.1	− 0.21
Potassium, mg/dL	668	4.0 ± 0.6	3.9 ± 0.6	0.10	3.9 ± 0.6	4.0 ± 0.6	− 0.08
Platelet, 1000/μL	662	182.9 ± 76.3	182.5 ± 81.0	0.01	179.9 ± 75.3	195.5 ± 87.0	− 0.19
Hematocrit, %	664	36.4 ± 7.3	35.9 ± 6.7	0.07	36.5 ± 7.4	35.9 ± 6.6	0.08
WBC, 1000/μL	663	10.2 ± 5.3	10.6 ± 5.1	− 0.07	9.7 ± 5.0	10.9 ± 4.8	− 0.25
Albumin, mg/dL	456	3.4 ± 0.5	3.3 ± 0.5	0.07	3.4 ± 0.5	3.2 ± 0.5	0.33
Lactate, mg/dL	263	28.6 ± 35.4	26.8 ± 20.5	0.07	27.7 ± 38.2	25.7 ± 20.5	0.07
INR	506	1.3 ± 0.4	1.4 ± 0.4	− 0.22	1.3 ± 0.4	1.3 ± 0.4	− 0.07

*PSM* propensity score matching, *STD* standard difference, *LVEF* left ventricular ejection fraction, *eGFR* estimated glomerular filtration rate, *AMI* acute myocardial infarction, *PCI* percutaneous coronary intervention, *IABP* intra-aortic balloon pumping, *ICU* intensive care unit, *SOFA* Sequential Organ Failure Assessment, *APACHE* Acute Physiology and Chronic Health Evaluation, *ACEi* angiotensin- converting enzyme inhibitor, *ARBs* angiotensin II receptor blockers, *MRA* mineralocorticoid receptor antagonist, *ALT* alanine aminotransferase, *BNP* B-type natriuretic peptide, *BUN* blood urea nitrogen, *NA* not available, *WBC* white blood count, *INR* international normalized ratio

^‡^Data were presented as number (%), mean ± standard deviation or median [25th, 75th percentile];

^*^Included in the calculation of propensity score

**Table 2:** Baseline characteristics of patients with eGFR < 30 or dialysis who received levosimendan versus dobutamine alone

Variable		Before PSM‡			After PSM‡		
	Valid N	Levosimendan (*n* = 52)	Dobutamine (*n* = 374)	STD	Levosimendan (*n* = 43)	Dobutamine (*n* = 81)	STD
Age, year*	426	72.6 ± 12.1	70.4 ± 11.9	0.18	72.6 ± 11.6	71.3 ± 11.3	0.11
Male*	426	31 (59.6)	237 (63.4)	− 0.08	23 (53.5)	51 (63.0)	− 0.19
LVEF, %*	426	25.9 ± 7.4	29.6 ± 7.1	− 0.51	26.4 ± 7.5	25.6 ± 8.1	0.10
eGFR, mL/min/1.73m^2^*	279	18.8 ± 6.7	19.1 ± 6.7	0.08	18.3 ± 6.6	21.1 ± 6.1	− 0.43
Prior dialysis*	426	18 (34.6)	129 (34.5)	0.00	15 (34.9)	30 (37.0)	− 0.04
Inotropic agents during the index admission							
Dopamine*	426	14 (26.9)	51 (13.6)	0.34	10 (23.3)	19 (23.5)	< 0.01
Norepinephrine*	426	10 (19.2)	77 (20.6)	− 0.03	9 (20.9)	18 (22.2)	− 0.03
Epinephrine*	426	5 (9.6)	59 (15.8)	− 0.19	4 (9.3)	9 (11.1)	− 0.06
AMI during the index admission*	426	16 (30.8)	85 (22.7)	0.18	14 (32.6)	22 (27.2)	0.12
PCI during the index admission*	426	11 (21.2)	36 (9.6)	0.32	9 (20.9)	16 (19.8)	0.03
Myocarditis during the index admission*	426	3 (5.8)	2 (0.5)	0.30	1 (2.3)	1 (1.2)	0.08
Mechanical ventilator*	426	36 (69.2)	151 (40.4)	0.61	27 (62.8)	46 (56.8)	0.12
IABP*	426	13 (25.0)	26 (7.0)	0.51	7 (16.3)	15 (18.5)	− 0.06
ICU days	426	11 [4, 18]	3 [0, 10]	NA	11 [4, 21]	5 [0, 13]	NA
Admission days	426	29 [15.5, 49]	24 [13, 42]	NA	29 [16, 53]	25 [13, 39]	NA
Risk score*							
SOFA	426	8.4 ± 2.1	8.9 ± 2.4	− 0.21	8.6 ± 2.2	8.6 ± 2.0	− 0.04
APACHE III	426	50.2 ± 13.3	49.8 ± 15.4	0.03	50.9 ± 13.8	49.4 ± 13.5	0.11
Comorbidities							
Atrial fibrillation	426	16 (30.8)	145 (38.8)	− 0.17	15 (34.9)	24 (29.6)	0.11
Diabetes mellitus	426	35 (67.3)	249 (66.6)	0.02	29 (67.4)	52 (64.2)	0.07
Hypertension	426	43 (82.7)	311 (83.2)	− 0.01	35 (81.4)	63 (77.8)	0.09
Dyslipidemia	426	35 (67.3)	223 (59.6)	0.16	28 (65.1)	57 (70.4)	− 0.11
Baseline medications							
Aspirin	426	43 (82.7)	250 (66.8)	0.37	35 (81.4)	61 (75.3)	0.15
Clopidogrel	426	35 (67.3)	214 (57.2)	0.21	28 (65.1)	44 (54.3)	0.22
Ticagrelor	426	18 (34.6)	72 (19.3)	0.35	12 (27.9)	22 (27.2)	0.02
Beta-blockers	426	46 (88.5)	325 (86.9)	0.05	38 (88.4)	67 (82.7)	0.16
ACE inhibitors	426	30 (57.7)	163 (43.6)	0.29	23 (53.5)	35 (43.2)	0.21
ARB	426	37 (71.2)	235 (62.8)	0.18	29 (67.4)	53 (65.4)	0.04
MRA	426	34 (65.4)	196 (52.4)	0.27	31 (72.1)	39 (48.1)	0.50
Digoxin	426	20 (38.5)	153 (40.9)	− 0.05	20 (46.5)	29 (35.8)	0.22
Amiodarone	426	30 (57.7)	188 (50.3)	0.15	27 (62.8)	33 (40.7)	0.45
Ivabradine	426	28 (53.8)	84 (22.5)	0.68	22 (51.2)	23 (28.4)	0.48
Baseline laboratory data							
Hemoglobin, g/dl	425	11.3 ± 2.7	10.0 ± 2.0	0.55	11.1 ± 2.7	10.5 ± 2.2	0.27
ALT, U/L	376	28 [15, 130]	23 [15, 45]	NA	35 [16, 130]	29 [16, 70]	NA
BNP, pg/mL	271	4634 [1625, 5000]	2412 [1231.9, 4700]	NA	4634 [1625, 5000]	3030 [1580, 4700]	NA
HCO3, mmol/L	261	21.1 ± 6.0	23.0 ± 5.8	− 0.31	21.2 ± 6.3	21.6 ± 5.0	− 0.08
Total bilirubin, mg/dL	288	1.6 ± 2.0	1.5 ± 2.1	0.05	1.6 ± 2.1	1.5 ± 2.0	0.06
BUN, mg/dL	419	66.7 ± 37.0	70.3 ± 38.6	− 0.10	70.6 ± 38.0	68.0 ± 36.7	0.07
Sodium, mg/dL	426	139.2 ± 6.5	139.2 ± 6.3	0.00	140.0 ± 6.8	139.4 ± 6.7	0.09
Potassium, mg/dL	426	4.2 ± 0.8	4.1 ± 0.7	0.06	4.3 ± 0.7	4.1 ± 0.7	0.17
Platelet, 1000/μL	424	163.7 ± 71.9	169.1 ± 85.4	− 0.07	166.3 ± 75.3	183.3 ± 92.1	− 0.20
Hematocrit, %	425	34.1 ± 7.6	30.7 ± 5.9	0.51	34.0 ± 7.6	32.1 ± 6.5	0.28
WBC, 1000/μL	424	9.9 ± 3.9	10.1 ± 5.5	− 0.04	10.4 ± 3.9	10.6 ± 5.0	− 0.05
Albumin, mg/dL	328	3.2 ± 0.5	3.1 ± 0.5	0.05	3.1 ± 0.5	3.2 ± 0.5	− 0.11
Lactate, mg/dL	184	29.9 ± 34.1	32.9 ± 32.5	− 0.09	30.5 ± 36.2	41.5 ± 39.9	− 0.29
INR	319	1.7 ± 1.0	1.6 ± 0.8	0.17	1.8 ± 1.0	1.5 ± 0.8	0.34

*PSM* propensity score matching, *STD* standard difference, *LVEF* left ventricular ejection fraction, *eGFR* estimated glomerular filtration rate, *AMI* acute myocardial infarction, *PCI* percutaneous coronary intervention, *IABP* intra-aortic balloon pumping, *ICU* intensive care unit, *SOFA* Sequential Organ Failure Assessment, *APACHE* Acute Physiology and Chronic Health Evaluation, *ACEi* angiotensin-converting enzyme inhibitor, *ARBs* angiotensin II receptor blockers, *MRA* mineralocorticoid receptor antagonist, *ALT* alanine aminotransferase, *BNP* B-type natriuretic peptide, *BUN* blood urea nitrogen, *NA* not available, *WBC* white blood count, *INR* international normalized ratio

^‡^Data were presented as number (%), mean ± standard deviation or median [25th, 75th percentile];

^*^Included in the calculation of propensity score

### ***Mortality rates in the eGFR*** ≥ ***30 mL/min/1.73 m***^***2***^*** subgroup***

**Fig. 2:**
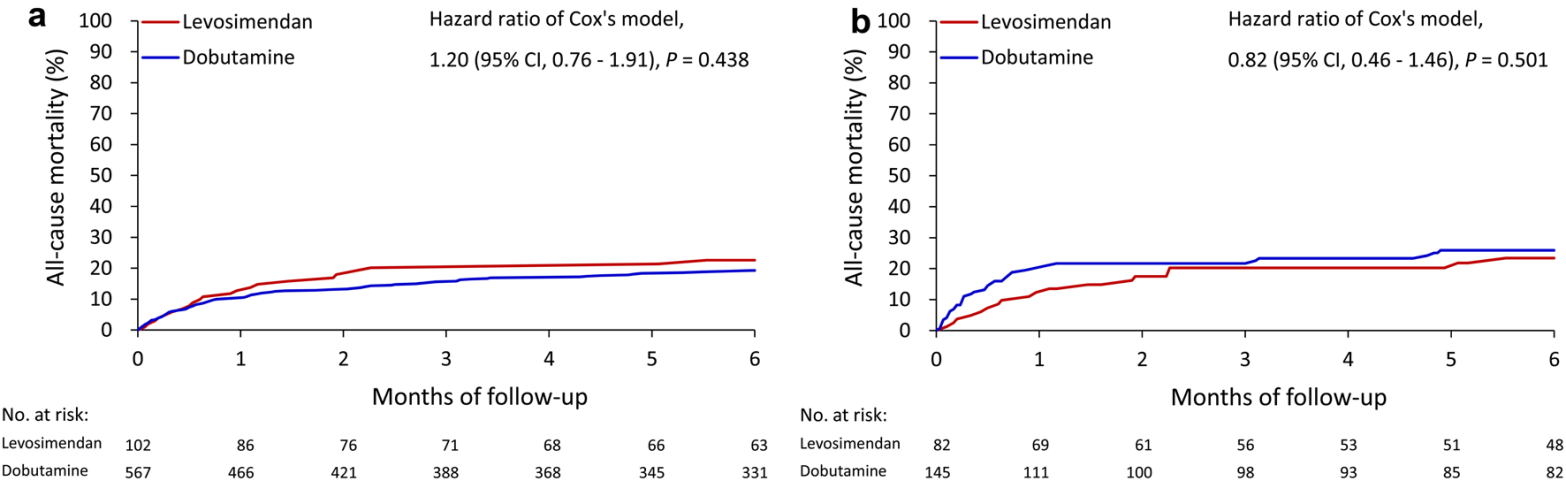
All-cause mortality of the patients with eGFR ≥ 30 mL/min/1.73 m^2^ who received levosimendan versus dobutamine alone before (**a**) and after (**b**) propensity score matching

For the patients with an eGFR ≥ 30 mL/min/1.73 m^2^, the overall in-hospital mortality rates were 12.7% in the levosimendan group and 10.8% in the dobutamine group [hazard ratio (HR) 0.80, 95% confidence interval (CI) 0.43–1.51]. The 30-day mortality rates were 12.7 and 10.1% in the levosimendan and dobutamine groups, respectively (HR 1.23, 95% CI 0.68–2.25). After PSM, the overall in-hospital mortality rates were 11.0% and 17.2% in the levosimendan and dobutamine groups, respectively (HR 0.37, 95% CI 0.15–0.92). The 30-day mortality rates were 12.2 and 19.3% in the levosimendan and dobutamine groups, respectively (HR 0.59, 95% CI 0.8–1.23). The differences in the 90- and 180-day mortality rates were nonsignificant between groups before or after PSM (Table 3; Fig. 2a, b).

**Table 3:** Mortality rates of patients with eGFR ≥ 30 who received levosimendan versus dobutamine alone

Outcomes	Event (%)		Levosimendan vs*.* dobutamine	
	Levosimendan	Dobutamine	HR (95% CI)	*P* value
Before PSM patient numbers	102	567		
In-hospital mortality	13 (12.7)	61 (10.8)	0.80 (0.43–1.51)	0.491
5-day mortality	3 (2.9)	19 (3.4)	0.87 (0.26–2.94)	0.822
14-day mortality	7 (6.9)	38 (6.7)	1.004 (0.45–2.25)	0.992
30-day mortality	13 (12.7)	57 (10.1)	1.23 (0.68–2.25)	0.496
90-day mortality	20 (19.6)	83 (14.6)	1.31 (0.80–2.13)	0.285
180-day mortality	22 (21.6)	99 (17.5)	1.20 (0.76–1.91)	0.438
After PSM patient numbers	82	145		
In-hospital mortality	9 (11.0)	25 (17.2)	0.37 (0.15–0.92)	0.032
5-day mortality	2 (2.4)	10 (6.9)	0.34 (0.07–1.60)	0.173
14-day mortality	5 (6.1)	19 (13.1)	0.44 (0.17–1.14)	0.091
30-day mortality	10 (12.2)	28 (19.3)	0.59 (0.28–1.23)	0.160
90-day mortality	16 (19.5)	31 (21.4)	0.85 (0.45–1.60)	0.604
180-day mortality	18 (22.0)	36 (24.8)	0.82 (0.46–1.46)	0.501

*HR* hazard ratio, *CI* confidence interval, *PSM* propensity score matching

### ***Mortality rates in the eGFR*** < ***30 mL/min/1.73m***^***2***^*** or dialysis subgroup***

**Fig. 3:**
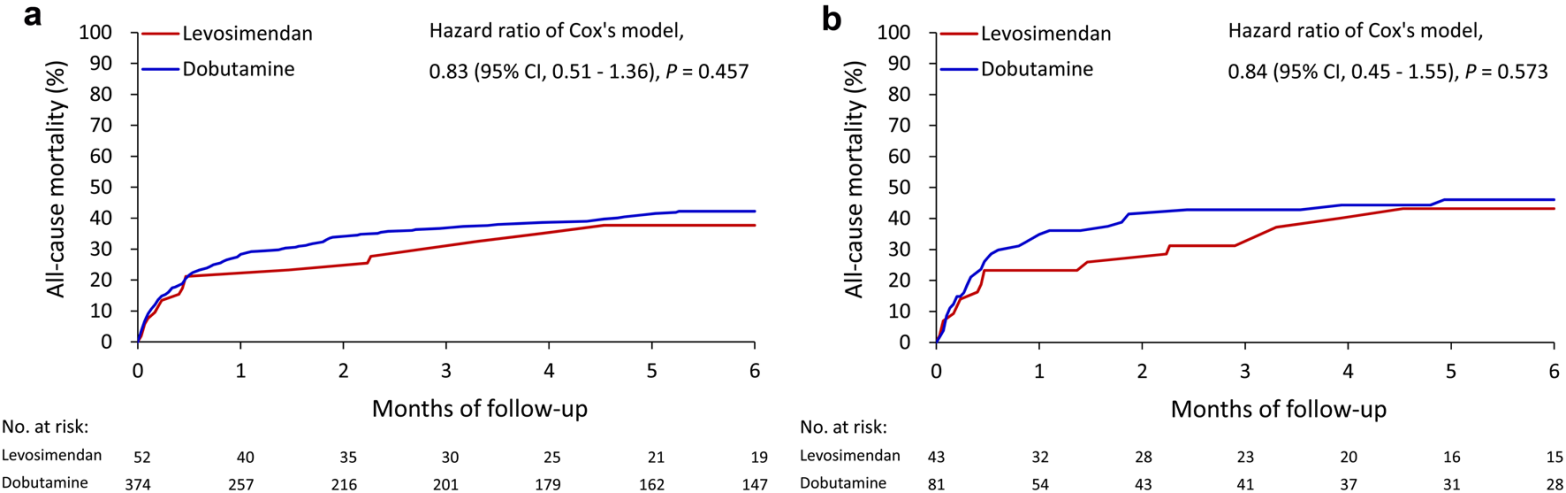
All-cause mortality rates of the patients with eGFR < 30 mL/min/1.73 m^2^ or dialysis who received levosimendan versus dobutamine alone before (**a**) and after (**b**) propensity score matching

For the patients with an eGFR < 30 mL/min/1.73 m^2^ or on maintenance dialysis, the overall in-hospital mortality rates were 26.9 and 30.5% in the levosimendan and dobutamine groups, respectively (HR 0.76, 95% CI 0.44–1.32). The 30-day mortality rates were 21.2 and 27.8% in the levosimendan and dobutamine groups, respectively (HR 0.73, 95% CI 0.39–1.37). After PSM, the overall in-hospital mortality rates were 30.2 and 37.0% in the levosimendan and dobutamine groups, respectively (HR 0.67, 95% CI 0.36–1.26). The 30-day mortality rates were 23.3 and 34.6% in the levosimendan and dobutamine groups, respectively (HR 0.65, 95% CI 0.30–1.37). The differences in the 90- and 180-day mortality rates were nonsignificant between the two groups before or after PSM (Table 4; Fig. 3a, b).

**Table 4:** Mortality rates of patients with eGFR < 30 or dialysis who received levosimendan versus dobutamine alone

Outcomes	Event (%)		Levosimendan vs. dobutamine	
	Levosimendan	Dobutamine	HR (95% CI)	*P* value
Before PSM patient numbers	52	374		
In-hospital mortality	14 (26.9)	114 (30.5)	0.76 (0.44–1.32)	0.330
5-day mortality	5 (9.6)	45 (12.0)	0.79 (0.31–1.98)	0.611
14-day mortality	11 (21.2)	76 (20.3)	1.01 (0.54–1.91)	0.968
30-day mortality	11 (21.2)	104 (27.8)	0.73 (0.39–1.37)	0.330
90-day mortality	14 (26.9)	132 (35.3)	0.72 (0.42–1.25)	0.247
180-day mortality	18 (34.6)	148 (39.6)	0.83 (0.51–1.36)	0.457
After PSM patient numbers	43	81		
In-hospital mortality	13 (30.2)	30 (37.0)	0.67 (0.36–1.26)	0.212
5-day mortality	4 (9.3)	10 (12.3)	0.76 (0.26–2.22)	0.611
14-day mortality	10 (23.3)	21 (25.9)	0.87 (0.42–1.80)	0.707
30-day mortality	10 (23.3)	28 (34.6)	0.65 (0.30–1.37)	0.254
90-day mortality	13 (30.2)	34 (42.0)	0.68 (0.35–1.31)	0.248
180-day mortality	17 (39.5)	36 (44.4)	0.84 (0.45–1.55)	0.573

*HR* hazard ratio, *CI* confidence interval, *PSM* propensity score matching

## Discussion

To the best of our knowledge, this is the first study to comprehensively investigate the short- and long-term survival of critical patients with both AHF and severe renal dysfunction who received levosimendan. We found that the patients who received levosimendan had similar survival rates to the patients who received dobutamine. Moreover, the differences in mortality rate were consistently nonsignificant between the two groups up to 180 days of follow-up, regardless of whether their eGFR was > 30 or < 30 mL/min/1.73 m^2^ or they were on maintenance dialysis.

Previous studies have reported that the use of levosimendan in patients with heart failure can improve cardiac output, urine amount, and eGFR. These results have been demonstrated in patients with acute or chronic left ventricular systolic dysfunction [6, 7, 10, 11, 17–20]. However, few studies have established the efficacy and safety of levosimendan in patients with severe renal dysfunction. Despite a lack of convincing evidence, depressed eGFR was thought to reduce the renal excretion of levosimendan leading to fatal arrhythmia, and several studies about levosimendan even excluded patients with an eGFR of < 30 mL/min/1.73 m^2^ [11, 21]. The Taiwan Food and Drug Administration (FDA) also does not recommend levosimendan for such patients. However, patients who are admitted for AHF frequently have different severities of renal dysfunction, and therefore the depressed GFR at that time could adversely affect the rate of levosimendan use. Although dobutamine may temporarily improve cardiac output, it can also lead to serious arrhythmia and myocardial ischemia [22, 23]. In contrast, the neutral effect on myocardial diastolic function and arrhythmogenic potential may suggest that levosimendan is a suitable alternative to dobutamine [24–26]. Levosimendan plays an important role in treating patients with severe renal dysfunction in our clinical practice.

In our CCUs, patients with acutely deteriorating heart failure are treated using diuretic agents, nitroglycerin, and ACEis (or angiotensin II antagonists) first, and we only prescribe inotropic agents for those with signs of systemic tissue hypoperfusion, including oliguria or a rapidly worsening plasma creatinine level. Because low cardiac output and systemic hypoperfusion in patients with reduced left ventricular systolic function is a critical situation, our intensivists are not limited with regard to the choice of treatment. It depends on their clinical judgement, including whether or not they combine inotropic agents with norepinephrine. The initial eGFR was ≥ 30 mL/min/1.73 m^2^ in most of our patients, but then became further depressed due to deteriorating cardiac function. We consider that renal dysfunction is reversible and not a contraindication for using levosimendan. For patients with an eGFR < 30 mL/min/1.73 m^2^, levosimendan is prescribed based on detailed discussions among the patients, their family members, and our critical medical teams in our clinical practice. The available medical evidence is discussed at these meetings [27–30], and the patients are carefully monitored to avoid adverse events. In this study, 53.9% of the patents in the levosimendan group received dopamine or dobutamine before the initiation of levosimendan in the index admission. This suggests that the patients were poor responders to dopamine or dobutamine. In our practice, some poor responders will receive mechanical cardiac support, such as veno-arterial ECMO or a temporary ventricular assist device (Centrimag), instead of levosimendan [34–36]. In the current study, none of the patients received a durable ventricular assist device or heart transplantation, and this situation is common in the medical environment in Taiwan.

The average LVEF of our study patients was < 30%. Before PSM, more patients in the levosimendan group had AMI and received PCI in the index admission, and dopamine and intra-aortic balloon pumps were more frequently used in this group. There was also a higher rate of myocarditis, however the difference was small. After PSM, the rates of AMI, PCI, mechanical ventilation, IABP, APACHE III, and SOFA scores between the two groups were similar. There were no significant differences in mortality rates up to 6 months between the two groups. In the subgroup with an eGFR < 30 mL/min/1.73 m^2^, the survival rate of the patients who received levosimendan was also equivalent to the patients who received dobutamine. Our results suggest that levosimendan did not increase mortality even in these critical AHF patients with severe renal dysfunction.

### Limitations

There are several limitations to this study. First, because of the retrospective and real-practice design, we could not limit our intensivists in their choices of inotropic agents. The prescription of levosimendan was based on the clinical judgement of the intensivists. For example, the rate of dopamine use was higher in the levosimendan group at baseline, so we used PSM to minimize bias. In total, 53.9% of the patients who received levosimendan were prescribed dobutamine or dopamine before their index date, and the outcomes may have been affected if the clinicians decided to continue dobutamine or dopamine at that time. These results can be only confirmed by well-designed prospective studies; however, it would be ethically wrong if the patients are considered to be poor responders to initial inotropic agents and clinicians do not adjust their therapeutic policies. Second, we excluded patients with ECMO implantation from our study. Veno-arterial ECMO is considered to be an emergency life-saving method for patients with AHF complicated with refractory cardiogenic shock. However, refractory cardiogenic shock is a contraindication for levosimendan. Moreover, the disease severity in the patients who needed ECMO was highly heterogeneous, ranging from post-cardiotomy shock to ECMO-assisted cardiopulmonary resuscitation for prolonged cardiac arrest. Therefore, differences in mortality rate between those who received levosimendan and dobutamine may have been affected by the use ECMO. Third, we used ICD codes to identify patients with heart failure and other comorbidities. This is common in database studies, however it can lead to misestimations [31, 32]. To minimize possible bias, we only enrolled patients who received inotropic agents and had a reduced ejection fraction, thereby reinforcing the diagnosis of heart failure. Fourth, we used pre-dosing plasma creatinine to estimate GFR. Because creatinine is not an instant parameter reflecting renal function, the baseline GFR may have been overestimated in this study. Collecting urine for 24 h increases the accuracy of GFR values; however, this cannot always be done for such critical patients.

## Conclusions

The critical AHF patients with or without severe renal dysfunction who received levosimendan had similar survival rates compared to the patients who received dobutamine while patients with veno-arterial ECMO were excluded. According to our results, an eGFR < 30 mL/min/1.73 m^2^ is not necessarily a contraindication for levosimendan.

## Data Availability

All data generated or analyzed during this study are included in this published article.

## Abbreviations

AHF: Acute heart failure
AMI: Acute myocardial infarction
CCU: Critical cardiac care unit
CKD: Chronic kidney disease
ECMO: Extracorporeal membrane oxygenation
eGFR: Estimated glomerular filtration rate
IABP: Intra-aortic balloon pump
PSM: Propensity score matching
LVEF: Left ventricular ejection fraction
PCI: Percutaneous coronary intervention
